# Advanced Composite Materials Utilized in FDM/FFF 3D Printing Manufacturing Processes: The Case of Filled Filaments

**DOI:** 10.3390/ma16186210

**Published:** 2023-09-14

**Authors:** Antreas Kantaros, Evangelos Soulis, Florian Ion Tiberiu Petrescu, Theodore Ganetsos

**Affiliations:** 1 Department of Industrial Design and Production Engineering, University of West Attica, 12244 Athens, Greece; 2 Theory of Mechanisms and Robots Department, Faculty of Industrial Engineering and Robotics, Bucharest Polytechnic University, 060042 Bucharest, Romania

**Keywords:** 3D printing, additive manufacturing, composite materials, filled filaments, FDM, FFM

## Abstract

The emergence of additive manufacturing technologies has brought about a significant transformation in several industries. Among these technologies, Fused Deposition Modeling/Fused Filament Fabrication (FDM/FFF) 3D printing has gained prominence as a rapid prototyping and small-scale production technique. The potential of FDM/FFF for applications that require improved mechanical, thermal, and electrical properties has been restricted due to the limited range of materials that are suitable for this process. This study explores the integration of various reinforcements, including carbon fibers, glass fibers, and nanoparticles, into the polymer matrix of FDM/FFF filaments. The utilization of advanced materials for reinforcing the filaments has led to the enhancement in mechanical strength, stiffness, and toughness of the 3D-printed parts in comparison to their pure polymer counterparts. Furthermore, the incorporation of fillers facilitates improved thermal conductivity, electrical conductivity, and flame retardancy, thereby broadening the scope of potential applications for FDM/FFF 3D-printed components. Additionally, the article underscores the difficulties linked with the utilization of filled filaments in FDM/FFF 3D printing, including but not limited to filament extrusion stability, nozzle clogging, and interfacial adhesion between the reinforcement and matrix. Ultimately, a variety of pragmatic implementations are showcased, wherein filled filaments have exhibited noteworthy benefits in comparison to standard FDM/FFF raw materials. The aforementioned applications encompass a wide range of industries, such as aerospace, automotive, medical, electronics, and tooling. The article explores the possibility of future progress and the incorporation of innovative reinforcement materials. It presents a plan for the ongoing growth and application of advanced composite materials in FDM/FFF 3D printing.

## 1. Introduction

Additive manufacturing, commonly referred to as 3D printing, involves the production of three-dimensional objects through the sequential deposition of material layers under the guidance of computerized instructions, known as gcode. It is a technology that has revolutionized the way that engineers perceive the concept of manufacturing and has the potential to change the modus operandi of various industry 4.0 processes [1,2,3,4,5].

Numerous 3D printing technologies exist, each possessing distinctive features and benefits [6]. Fused Deposition Modeling (FDM)/Fused Filament Fabrication (FFF) is a prevalent and extensively utilized technique for generating three-dimensional objects. This method involves the melting of a thermoplastic material and its subsequent extrusion through a nozzle [7,8]. The aforementioned technology is characterized by its affordability and frequent utilization in the creation of prototypes and limited production runs. It is important to note that Fused Deposition Modeling (FDM) is a proprietary term that has been trademarked by Stratasys, whereas Fused Filament Fabrication (FFF) is an open-source term that has been adopted by the RepRap community. The primary distinction between Fused Deposition Modeling (FDM) and Fused Filament Fabrication (FFF) 3D printing lies in the utilization of a thermoplastic filament that is heated and extruded through a nozzle in the former, whereas the latter employs a comparable process but with a broader spectrum of materials, encompassing metals, woods, and ceramics. According to the published literature [9,10], FFF printers are characterized by a higher degree of customization options, which enable users to fine-tune print speed, temperature, and layer height. Conversely, FDM printers are generally considered more user-friendly and require less setup time.

Stereolithography (SLA) is a prevalent 3D printing technology that utilizes a liquid resin that undergoes curing by a laser to fabricate a three-dimensional object. The aforementioned technology has garnered a reputation for its exceptional precision and accuracy, rendering it well-suited for the production of intricate and elaborate objects [11,12,13]. Selective Laser Sintering (SLS) is a 3D printing methodology that utilizes a laser to selectively bond powdered materials, leading to the creation of a three-dimensional entity. The technology mentioned above is commonly utilized for the production of components that possess strong mechanical characteristics, extended durability, and exceptional thermal stability. As a result, it is a feasible alternative for the creation of functional prototypes and end-use products, as documented in references [14,15,16].

The utilization of 3D printing technology offers numerous benefits when compared to conventional manufacturing techniques. The aforementioned technology exhibits exceptional velocity, efficacy, and economic viability, making it exceedingly appropriate for prototyping and limited-scale manufacturing. In addition, it promotes enhanced adaptability in the design process, thus allowing for the creation of complex and unique objects that would be difficult or impractical to produce using traditional manufacturing methods [17,18,19,20,21,22,23].

The utilization of 3D printing technology has been on the rise in various sectors, including healthcare, aerospace, automotive, and architecture. The healthcare sector employs 3D printing technology to manufacture prosthetics, implants, and surgical guides [24,25,26,27,28,29]. The application of this technology is widely observed in the aerospace sector for the manufacturing of complex engine components and structures designed for aerospace purposes [30,31,32]. Additive manufacturing technology is employed in the automotive sector to manufacture prototypes and final products [33,34,35]. The application of 3D printing within the field of architecture is widespread, primarily for the purpose of producing models for architectural design, prototyping, and constructing full-scale buildings [36,37,38].

The raw materials used in FFF/FDM 3D printing are typically filament-based materials that are either thermoplastics or thermoplastic composites. The raw materials exhibit diverse physical configurations, such as spools, pellets, and granules. The primary attribute of the raw materials employed in Fused Filament Fabrication (FFF) or Fused Deposition Modeling (FDM) 3D printing is their respective melting points. Since FFF/FDM 3D printing involves melting the raw materials, the melting point is a crucial factor that determines the printing temperature. The melting points of frequently utilized FFF/FDM materials, including ABS, PLA, and PETG, typically fall within the range of 170 °C to 250 °C. The melting point of the material determines how easily it can be melted and extruded through the printer nozzle, and also affects the strength and durability of the final printed object [39,40,41,42,43,44].

The second characteristic of FFF/FDM raw materials is their viscosity. Viscosity is a measure of the material’s resistance to flow, and it determines how easily the material can be extruded through the printer nozzle. FFF raw materials with higher viscosities require higher temperatures and pressure to extrude, which can lead to issues such as nozzle clogging, stringing, and warping. Lower-viscosity materials, on the other hand, can flow more easily and can produce smoother and more detailed prints. Materials such as ABS and Nylon have higher viscosities compared to materials like PLA and PETG [45,46,47].

**Figure 1:**
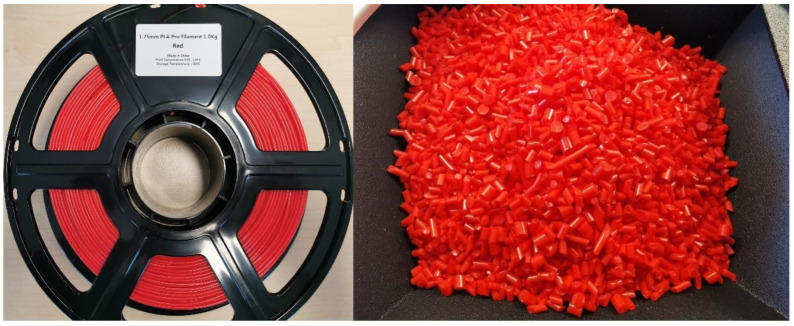
PLA raw material in the form of filament (**left**) and in the form of pellets (**right**).

The determination of the quality and accuracy of the printed object in FFF/FDM 3D printing is contingent upon the diameter of the raw materials utilized. The prevalent Fused Filament Fabrication (FFF) or Fused Deposition Modeling (FDM) materials are typically available in standard diameters of 1.75 mm and 2.85 mm, with some minor deviations in between. The extrusion rate of the filament and the printing speed, as well as the amount of material deposited per unit length, are influenced by the diameter of the raw material. The precision and exactness of the printed entity are also influenced by the diameter of the filament. Filaments with smaller diameters, such as 1.75 mm, are typically characterized by greater precision and accuracy when compared to filaments with larger diameters, such as 2.85 mm. The reason for this phenomenon is that filaments with smaller diameters necessitate less force to be exerted through the printer nozzle, thereby reducing material deformation and promoting uniform extrusion. Moreover, filaments with a reduced diameter possess a diminished cross-sectional area, thereby facilitating the production of intricate features and more defined contours in the printed artifact. Maintaining filament diameter consistency across the entire spool is crucial, as any fluctuations in diameter can result in irregular extrusion, ultimately compromising the quality of the printed output [48,49,50,51]. The first illustration, denoted as Figure 1, portrays the raw material of PLA in two distinct forms: filament on the left and pellets on the right.

PLA, ABS, PETG, and TPU are among the frequently utilized materials in Fused Filament Fabrication (FFF) or Fused Deposition Modeling (FDM) 3D printing. Distinctive properties and attributes are inherent in every material, rendering it appropriate for diverse purposes. To begin with, PLA, also known as Polylactic Acid, is a thermo-plastic material that is capable of undergoing biodegradation. This material is derived from renewable resources, including but not limited to cornstarch, sugarcane, and cassava. The printing process is facilitated by its ease of printability, low melting threshold, and minimal susceptibility to warping and shrinkage. PLA is a popular choice for 3D printing because it is environmentally friendly and produces a high-quality surface finish. ABS (Acrylonitrile Butadiene Styrene) is a thermoplastic polymer renowned for its robustness, pliability, and capacity to withstand impact. This material is widely used in various applications due to its exceptional mechanical properties. It is also relatively inexpensive and has good thermal and chemical resistance [52]. The Acrylonitrile Butadiene Styrene (ABS) material is frequently employed in various industrial sectors that necessitate robustness and endurance, including but not limited to automotive components, playthings, and electronic casings [53,54]. Also, PETG (Polyethylene Terephthalate Glycol) is a thermoplastic material that combines the best properties of both PLA and ABS. It has good strength and durability, is easy to print, and has good chemical and impact resistance. PETG is a popular choice for 3D printing because it is food safe and produces a glossy finish. It is also suitable for outdoor applications due to its UV resistance [55]. In addition, TPU (Thermoplastic Polyurethane) is a flexible and elastic material that is commonly used for applications that require flexibility and impact resistance, such as phone cases, toys, and shoe soles. According to reference [56], TPU exhibits resistance to oil, grease, and abrasion.

Another greater material category that can be utilized as raw materials in FDM/FFF 3D printing technology are the so called “filled” materials. They also come in a filament form like the aforementioned materials but feature key differences. The main difference is that filled material filaments are a type of 3D printing material that contain additives or fillers that improve their mechanical, thermal, or aesthetic properties. In this context, these materials feature a polymeric matrix and another material such as an additive or filler, therefore categorizing them as composite materials. There are several categories of filled material filaments, each with its own unique set of properties and advantages [57,58].

Metal-filled filaments constitute a category of filled material filaments, wherein a polymer base is blended with fine metal powder [59]. The aforementioned filaments are capable of generating components that exhibit enhanced thermal and electrical conductivity, as well as a metallic surface finish. These materials are appropriate for use in contexts that demand elevated levels of potency, longevity, and thermal resilience, such as the manufacturing of automobile components and aerospace constituents. A distinct classification pertains to carbon fiber-infused filaments that encompass a polymer base blended with either chopped or continuous carbon fibers [60]. The utilization of said filaments results in the production of components characterized by elevated levels of rigidity, potency, and reduced mass. These materials exhibit favorable strength-to-weight ratios, rendering them appropriate for deployment in contexts that demand such attributes, such as unmanned aerial vehicles, automated machines, and artificial body parts. Glass fiber-filled filaments are a type of filled material filament that comprises a polymer base mixed with chopped or continuous glass fibers [61]. The aforementioned filaments have the ability to generate components that exhibit elevated levels of strength, stiffness, and dimensional stability. These components are well-suited for utilization in scenarios that necessitate precise measurements and consistent dimensions, such as engineering prototypes and parts. Wood-filled filaments are a type of filled material filament that comprise a polymer base blended with wood particles or fibers. The utilization of said filaments results in the production of components that exhibit a wood-like aesthetic and tactile quality, rendering them appropriate for employment in decorative or artistic contexts. According to the published literature findings [62], these products exhibit eco-friendliness and biodegradability.

Optimal printing conditions for filled filaments, which incorporate additives like metals, carbon fiber, or wood, can vary based on the material composition. Generally, carbon fiber-filled filaments benefit from nozzle temperatures in the range of 230 °C to 260 °C due to the abrasive nature of carbon fiber. Metal-filled filaments, such as bronze, copper, or stainless steel, usually print well between 195 °C and 230 °C. Wood-filled filaments perform best at temperatures ranging from 175 °C to 220 °C to maintain a wood-like appearance. While chamber temperature is less critical than nozzle temperature, maintaining a chamber temperature between 40 °C and 60 °C can help improve adhesion and reduce warping for filled filaments. However, these guidelines should be adapted to the specific filament brand and composition, while experimentation may be necessary to achieve optimal print quality on each specific 3D printer.

Overall, filled material filaments offer many advantages over standard filaments, including improved strength, stiffness, thermal and electrical conductivity, dimensional stability, and aesthetic properties. They are suitable for a wide range of applications in various industries, such as automotive, aerospace, engineering, and art. It is important to select the appropriate filled material filament for the specific requirements of the application to ensure the best results.

## 2. Materials

### 2.1. Wood-Filled PLA

Wood-filled PLA is a composite material that combines the biodegradable thermoplastic PLA (Polylactic Acid) with wood fibers or particles. The wood content can range from finely ground wood flour to larger wood chips, depending on the desired appearance and mechanical properties. Wood-filled PLA offers a unique solution for 3D printing enthusiasts and professionals who seek to add a natural and organic touch to their printed objects [63,64,65,66].

**Figure 2:**
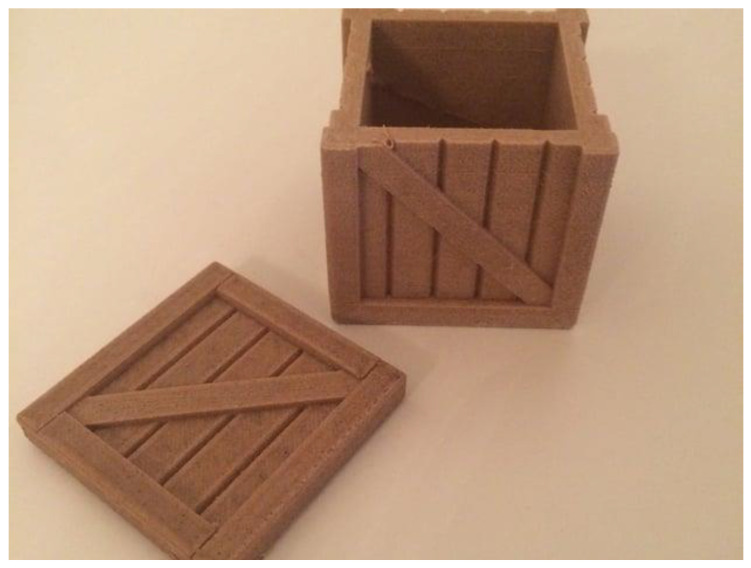
Item printed with wood-filled filament [67].

The addition of wood fibers or particles provides Wood-filled PLA with distinctive characteristics. First and foremost, it imparts a visually appealing wood-like texture and appearance to the printed objects. The presence of wood particles creates a natural grain pattern and surface roughness that can mimic the aesthetic qualities of real wood. This makes Wood-filled PLA an excellent choice for applications where the look and feel of wood are desired but the advantages of 3D printing, such as customization and intricate designs, are also sought. Figure 2 depicts an item printed with wood-filled filament [67].

Furthermore, Wood-filled PLA is known to exhibit varied mechanical properties compared to pure PLA filaments. The presence of wood particles introduces interruptions in the polymer bonds, resulting in a decrease in stiffness and structural integrity of the printed objects. As a result, Wood-filled PLA is more commonly utilized in applications such as artistic sculptures, architectural models, and various decorative items, rather than functional prototypes. It is important to note that the material may pose challenges during the printing process. Achieving proper layer adhesion can be problematic, and it generally necessitates extensive experimentation with slicer settings to mitigate issues such as clogging and stringing [68,69,70].

Applications for Wood-filled PLA can be found in a wide range of industries. In the field of product design, this filament can be utilized as raw materials to create furniture prototypes, custom handles, or decorative trims. In the arts and crafts domain, Wood-filled PLA enables the production of sculptures, figurines, or jewelry with a wood-like appearance. Additionally, in the architectural field, it can be utilized to fabricate scaled models, building components, or even entire structures with an organic touch. By harnessing the benefits of Wood-filled PLA, makers and designers can explore the intersection between the natural look of wood and the innovative capabilities of 3D printing technology, opening up new creative possibilities [71,72].

### 2.2. Metal-Filled Filaments

Metal-infused filaments represent a noteworthy category of raw materials that combine the flexibility of 3D printing with the advanced mechanical properties of metal. The filaments are composed of a substrate material, such as PLA or ABS, that has been infused with finely ground metallic particles. The inclusion of metallic elements such as copper, bronze, or stainless steel confers unique metallic attributes to the printed articles, encompassing their visual appeal, mass, and even their magnetic properties [73,74].

Metal-filled filaments possess the capability of replicating the gleaming visual appeal of conventional metals. The printed items exhibit an alluring metallic luster that arrests the viewer’s attention with their polished exteriors and reflects light with a captivating radiance. The aesthetic appeal of metal-infused filaments renders them a desirable option for ornamental purposes, wherein the aim is to emulate the opulent appearance of metals while circumventing the constraints of conventional manufacturing techniques.

Metal-infused filaments possess superior mechanical characteristics that distinguish them from standard filaments, in addition to their visual allure. The inclusion of metallic particles serves as a means of enhancing the mechanical behavior of the printed objects, specifically their strength, stiffness, and durability. The aforementioned characteristics render them highly appropriate for the development of functional prototypes, mechanical components, and even industrial parts of limited scale that require durability and longevity [75,76].

Nevertheless, it is imperative to take into account that the utilization of metal-infused filaments for printing entails a distinct array of difficulties. The printing process parameters require meticulous attention due to the high density and thermal conductivity exhibited by metals. To achieve successful outcomes, it is crucial to prioritize the proper setting of optimal extrusion temperatures, print speeds, and cooling mechanisms. In addition, supplementary post-processing procedures may be necessary to enhance the surface texture and attain the intended metallic attributes.

**Figure 3:**
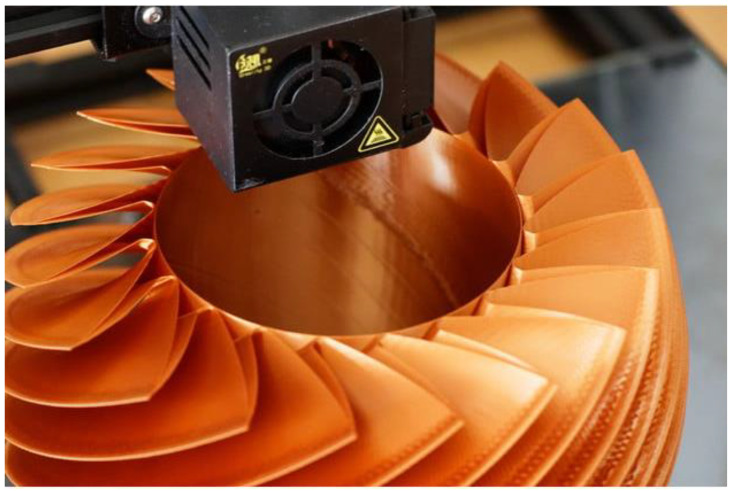
Image of 3D-printed items made out of metal-filled filaments [79].

Metal-filled filaments have a wide range of applications that are utilized across various industries. In the fields of engineering and manufacturing, the utilization of advanced techniques facilitates the production of functional prototypes, jigs, and fixtures that exhibit properties closely resembling those of metal parts. Metal-filled filaments are a viable and economical substitute for conventional casting techniques in the field of jewelry design. This alternative approach enables the production of intricate and personalized designs. Furthermore, the magnetic characteristics of specific metal-infused filaments present potential opportunities for utilization in the fields of electromagnetics, sensors, and educational exhibitions [77,78]. Figure 3 depicts an item being 3D printed out of metal filled filament.

To sum up, the utilization of metal-infused filaments offers an opportunity to combine the adaptability of 3D printing technology with the enduring appeal of metallic materials. These filaments present a compelling option for individuals interested in exploring the world of metallic 3D-printed objects due to their captivating appearance, enhanced mechanical properties, and diverse range of potential applications.

### 2.3. Carbon Fiber-Filled Filaments

Carbon fiber-filled filaments combine a base material, such as PLA or ABS, with carbon fiber strands or particles, resulting in printed objects that exhibit remarkable mechanical properties and a unique aesthetic appeal. One of the defining characteristics of carbon fiber-filled filaments is their exceptional strength-to-weight ratio. The incorporation of carbon fibers imparts outstanding stiffness and tensile strength to the printed objects, making them ideal for applications where lightweight yet robust components are essential. The carbon fiber reinforcement creates a structural integrity that surpasses traditional filaments, enabling the production of functional prototypes, aerospace components, automotive parts, and sporting equipment that demand high performance [80,81].

Beyond their mechanical properties, carbon fiber-filled filaments offer a distinct visual allure. The printed objects showcase a strikingly sleek and textured surface, accentuated by the visible carbon fiber patterns. The aesthetic attribute of the finished pieces confers a sense of refinement and contemporaneity, rendering them especially coveted for employment in the domains of design, architecture, and consumer goods [82].

While printing with carbon fiber-filled filaments presents advantages, it also presents certain challenges. Due to the abrasive nature of carbon fibers, specialized nozzles and extruders are often recommended to prevent excessive wear. Furthermore, attaining the ideal outcomes necessitates the identification of an appropriate equilibrium among printing temperature, printing speed, and cooling. Nevertheless, through meticulous modifications and empirical testing, the benefits in relation to mechanical efficiency and aesthetic attractiveness can be exceedingly noteworthy [83,84].

**Figure 4:**
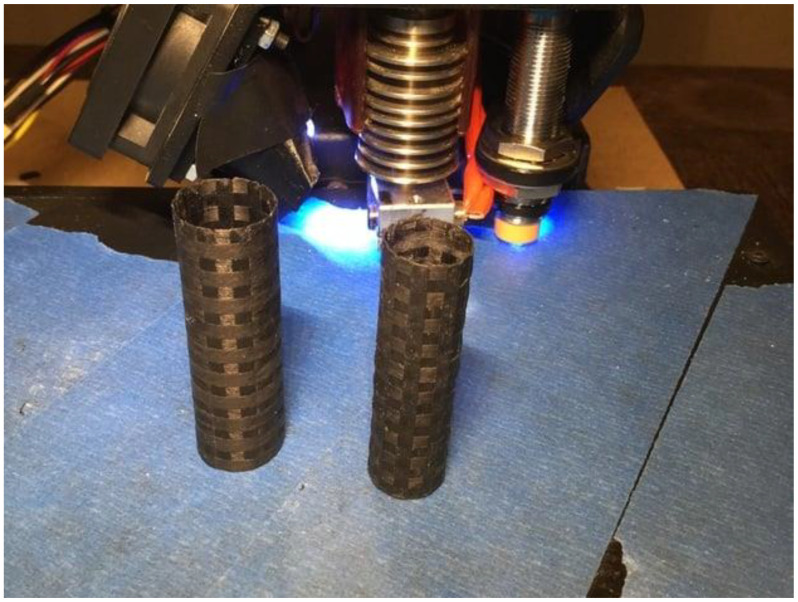
An image of 3D-printed items made out of a carbon fiber-filled filament.

The numerous and varied uses of filaments infused with carbon fiber are plentiful. Filaments have the potential to be utilized in the automotive industry for the purpose of manufacturing lightweight components such as custom accessories, engine parts, and interior trim. This can be achieved without compromising their structural integrity and safety. The incorporation of filaments infused with carbon fiber in the field of robotics enables the creation of durable and intricately designed structural elements. Furthermore, in the realm of sports and recreation, these fibers are employed in the production of bicycle frames, drone parts, and musical instruments, enhancing effectiveness while decreasing weight [85,86]. Figure 4 illustrates a 3D-printed item made out of a carbon fiber-filled filament.

In this context, the utilization of carbon fiber-filled filaments provides an avenue for exploiting the advanced mechanical and low-weight properties of carbon fiber in the context of 3D printing. The combination of their advanced mechanical capabilities and visually appealing design makes them a highly desirable option for end-users looking to innovate in diverse fields, by exploring the limits of both form and function.

### 2.4. Glass-Filled Filaments

Glass-filled filaments have advanced mechanical properties by incorporating glass fibers into a base material, typically PLA or ABS, for 3D printing. These filaments lever-age the reinforcing properties of glass fibers to enhance the mechanical characteristics of the printed objects.

The addition of glass fibers to the filament composition imparts remarkable strength and rigidity to the printed parts. The fibers create a reinforcing network within the material, effectively fortifying the structure and providing increased resistance to bending, torsion, and impact. These properties make glass-filled filaments ideal for applications that demand robustness, such as functional prototypes, industrial components, or mechanical parts subjected to higher stress levels [87,88].

In contrast to transparent and translucent filaments, glass-filled filaments do not possess transparency properties. Instead, the glass fibers contribute to the mechanical integrity of the printed objects. By optimizing the fiber content and distribution, engineers can achieve a desired balance between strength and printability, ensuring successful outcomes while harnessing the reinforcement benefits of glass fibers [89].

**Figure 5:**
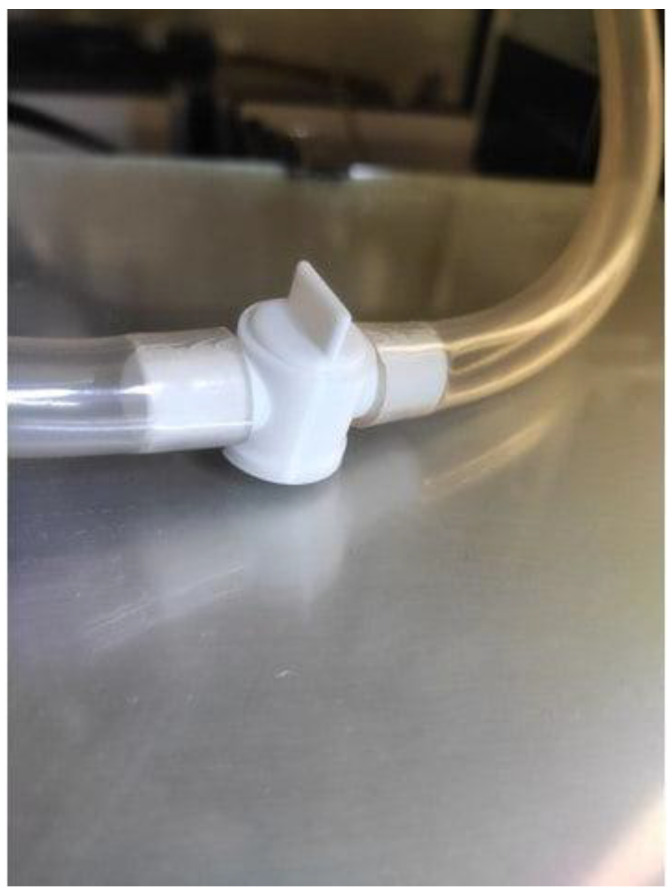
An image of 3D-printed items made out of glass-filled filament.

Working with glass-filled filaments does present certain considerations. The presence of glass fibers in the material can increase the wear on the printer nozzle, necessitating regular maintenance and potential nozzle upgrades. The careful calibration of printing parameters, including temperature, extrusion rate, and cooling, is crucial to achieve optimal results and mitigate issues such as clogging or warping [90,91]. Figure 5 depicts a 3D-printed item made out of a glass-filled filament.

The applications of glass-filled filaments span across various industries. In engineering and manufacturing, these filaments are favored for creating robust functional prototypes, jigs, fixtures, and even end-use parts where strength and durability are paramount. They find utility in industries such as automotive, aerospace, and electronics, where reliability and mechanical performance are crucial.

### 2.5. Conductive Filaments

Conductive filament represents a novel class of 3D printing raw materials that exhibit electrical conductivity while retaining the versatility of 3D printing technology. These filaments incorporate conductive additives, such as carbon nanotubes or graphene, into a base polymer matrix, enabling the printed objects to conduct electricity. This unique property opens up a wide range of possibilities in various fields, including electronics, sensors, and wearable devices [92,93].

The integration of conductive additives within the filament matrix facilitates the formation of a conductive pathway throughout the printed object. This pathway enables the flow of electrical current, allowing for the transmission of signals, the generation of heat, or the sensing of environmental parameters. The ability of inherent conductivity in 3D-printed objects with inherent conductivity offers great potential for the fabrication of custom electronic components, smart devices, and even integrated circuits [94,95].

The conductivity of these filaments can be tailored by adjusting the concentration and distribution of conductive additives. Higher loading levels of conductive particles generally result in increased electrical conductivity. However, careful control of the dispersion and alignment of the additives is crucial to ensure consistent and reliable conductivity throughout the printed object. Additionally, optimizing printing parameters, such as nozzle temperature and extrusion speed, is essential to maintain the structural integrity of the printed part while preserving its electrical properties [96].

The applications of conductive filaments are numerous and diverse. In the field of electronics, it enables the creation of flexible circuits, connectors, and antennas that can be seamlessly integrated into 3D-printed structures. These filaments also find utility in the development of sensors for detecting and monitoring various parameters, including temperature, strain, or touch. Furthermore, conductive filament paves the way for the production of wearable devices, such as smart textiles or personalized medical sensors, which benefit from the combination of electrical functionality and the freedom of design afforded by 3D printing [97].

**Figure 6:**
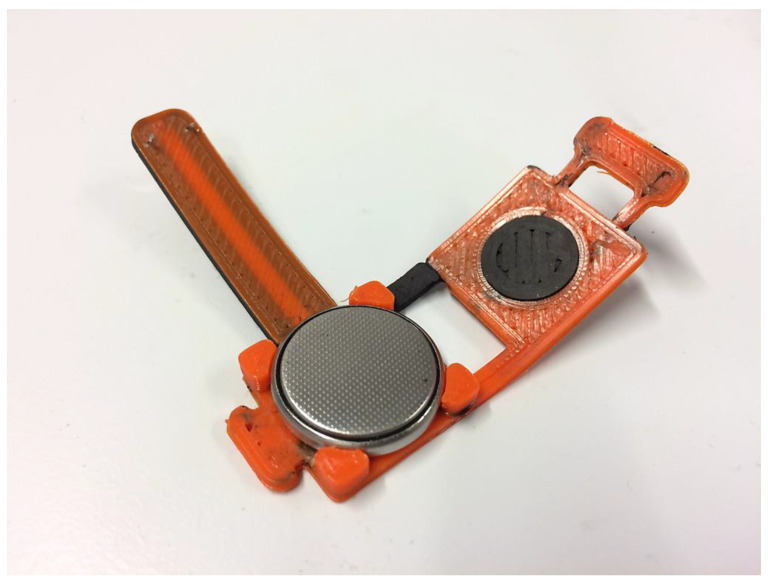
An image of 3D-printed items made out of conductive filament [100].

Despite the potential benefits of conductive filament, there are still obstacles to overcome in order to achieve optimal levels of electrical conductivity, consistency, and printability. Researchers are persistently investigating innovative formulations and manufacturing methodologies to augment the conductivity of the aforementioned filaments and ameliorate their performance attributes. Furthermore, progress in post-processing techniques, such as annealing or surface modifications, present prospects for further augmenting the electrical characteristics of printed entities [98,99]. Figure 6 shows a 3D-printed item made out of conductive material.

Therefore, the utilization of conductive filament presents an intriguing opportunity to integrate electrical conductivity within 3D-printed items. Through the integration of conductive additives with the underlying polymer matrix, these filaments facilitate the achievement of operational electronic components and devices. As the research and development process continues, the conceivable uses for conductive filament are anticipated to broaden, fundamentally transforming domains that are dependent on electronics and 3D printing technology.

### 2.6. Flexible TPU/TPE (Thermoplastic Elastomer)-Filled Filaments

Thermoplastic Elastomer (TPE) filaments are a noteworthy category of materials that combine the elasticity and pliability of elastomers with the advanced capabilities of additive manufacturing. The filaments are composed of a combination of polymers, which commonly comprise elastomeric materials like styrene–butadiene–styrene (SBS) or thermoplastic polyurethane (TPU). These materials possess distinctive mechanical characteristics that facilitate the creation of objects with exceptional elasticity and resilience.

The principal characteristic of thermoplastic elastomer (TPE) filaments that are flexible is their ability to undergo substantial deformation without causing permanent damage. The possession of a distinctive molecular configuration enables the polymer chains to readily undergo sliding and reorganization when subjected to external force, thereby conferring exceptional properties of elongation and recovery. The exceptional ability of flexible filaments to withstand bending, flexing, and impact absorption makes them ideal for deployment in various applications, including wearable devices, gaskets, and flexible joints [101,102,103].

Filled TPU (Thermoplastic Polyurethane) and TPE (Thermoplastic Elastomer) filaments represent a newly introduced intersection of flexibility and versatility in 3D printing materials. These materials are renowned for their rubber-like properties, including excellent elasticity, impact resistance, and durability. What sets filled TPU and TPE apart is the incorporation of additives or fillers into the base polymer to enhance specific attributes.

The rheological behavior of flexible filaments in 3D printing plays a significant role in determining their processability. This behavior is characterized by parameters such as viscosity, elasticity, and shear thinning. The extrusion process is influenced by various factors that necessitate the proper setting of the printer parameters, including nozzle temperature and print speed, in order to attain the desired outcome. Furthermore, it is imperative to optimize the adhesion of the printer bed and support structures in order to mitigate the risk of deformation or warping during the printing process [104,105,106]. For instance, carbon fiber-filled TPU/TPE offers increased stiffness and strength, making it suitable for applications that demand both flexibility and structural integrity, such as drone components or functional prototypes. On the other hand, TPU/TPE filaments infused with softer materials like thermoplastic elastomers can provide an even higher degree of flexibility, ideal for producing comfortable wearables, soft grips, and shock-absorbing components.

Such filaments can be applied across various fields, including robotics, prosthetics, and soft robotics. In robotics, these filaments enable the fabrication of flexible grippers, compliant actuators, and sensor integration, enhancing the dexterity and adaptability of robotic systems. In the field of prosthetics, flexible filaments offer the potential to create personalized and functional prosthetic components that can conform to the user’s anatomy and provide natural movement [107,108]. Another notable variant is the addition of conductive additives, which impart electrical conductivity to the material. This is valuable for creating custom, flexible circuitry, capacitive touch sensors, or EMI shielding solutions. The versatility of filled TPU and TPE filaments makes them prized assets in industries like healthcare, automotive, consumer electronics, and beyond, where the demand for both flexibility and specialized properties continues to grow. Moreover, the medical industry benefits from flexible filaments for the production of medical devices, such as surgical guides or anatomical models, which require a balance of flexibility and accuracy. Flexible filaments are integral to the development of soft, bio-inspired robots that can safely interact with humans and navigate intricate surroundings within the realm of soft robotics [109].

**Figure 7:**
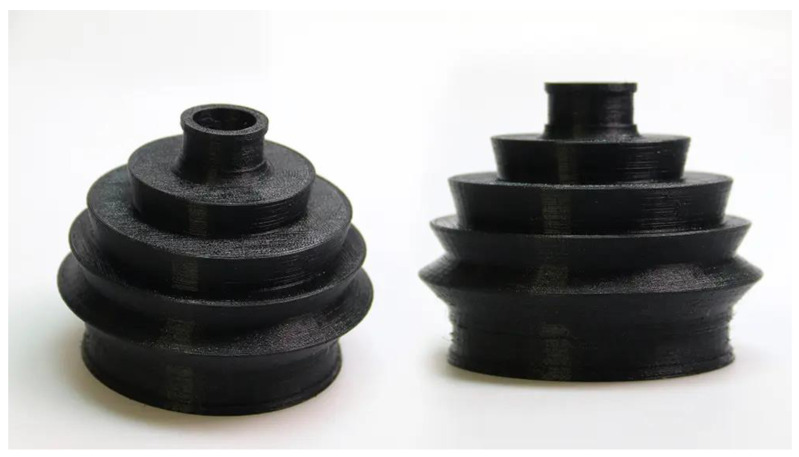
An image of 3D-printed items made out of flexible TPU-filled filament.

Ongoing research and development endeavors are being undertaken in the field of 3D printing to enhance the mechanical properties and printability of flexible filaments, and material alternatives to them. The applications of fabricating flexible 3D-printed objects are being expanded through innovations in material formulations and modifications, which may involve the incorporation of additives or blending with other polymers [110,111]. Figure 7 depicts 3D-printed items made out of filled flexible filament.

In this context, the utilization of filled TPU/TPE filaments presents a compelling opportunity for the manufacturing of objects that possess exceptional elasticity, resilience, and adaptability. These filaments possess distinctive mechanical properties that facilitate progress in the fields of robotics, prosthetics, and soft robotics, thereby expanding the horizons for applications that necessitate both flexibility and durability. As formulation and processing techniques are further developed by researchers, the potential for flexible filaments in the field of additive manufacturing is expected to expand. This expansion will facilitate the creation of increasingly intricate and practical printed objects.

### 2.7. Ceramic-Filled Filaments

Ceramic-filled filaments represent a category of 3D printing raw materials that integrate the adaptability of thermoplastic filament raw materials with the outstanding characteristics of ceramics. The filaments are composed of a matrix of thermo-plastic polymer that has been infused with ceramic particles of a small size. Filaments that are filled with ceramic materials provide benefits such as exceptional mechanical strength, electrical-insulation properties, and resistance to high temperatures.

Ceramic-filled filaments possess a notable benefit of exhibiting high-temperature resistance, rendering them appropriate for utilization in aerospace, automotive, and industrial domains. The presence of ceramic particles serves as a means of thermal insulation, thereby endowing the printed objects with the ability to withstand high temperatures without experiencing substantial deterioration [112].

Ceramic-infused filaments not only exhibit exceptional thermal stability but also augment the mechanical characteristics of the printed items. Ceramic materials are known for their hardness, rigidity, and abrasion resistance, transferring these qualities to the printed components. This makes ceramic-filled filaments suitable for applications that demand durability and precision [113,114,115].

However, it is important to note that ceramic-filled filaments can be brittle. The presence of ceramic particles can decrease the overall flexibility of the printed objects, making them more prone to breakage under certain conditions. Care must be taken to design and print components that can withstand mechanical stresses.

Printability can also be a challenge with ceramic-filled filaments due to the abrasive nature of ceramics. Regular maintenance and potential nozzle upgrades are necessary to mitigate increased wear on the printer nozzle. Furthermore, optimizing print settings, such as temperature, extrusion rate, and cooling, is crucial to ensure successful prints while preserving the mechanical and thermal properties of the ceramics.

**Figure 8:**
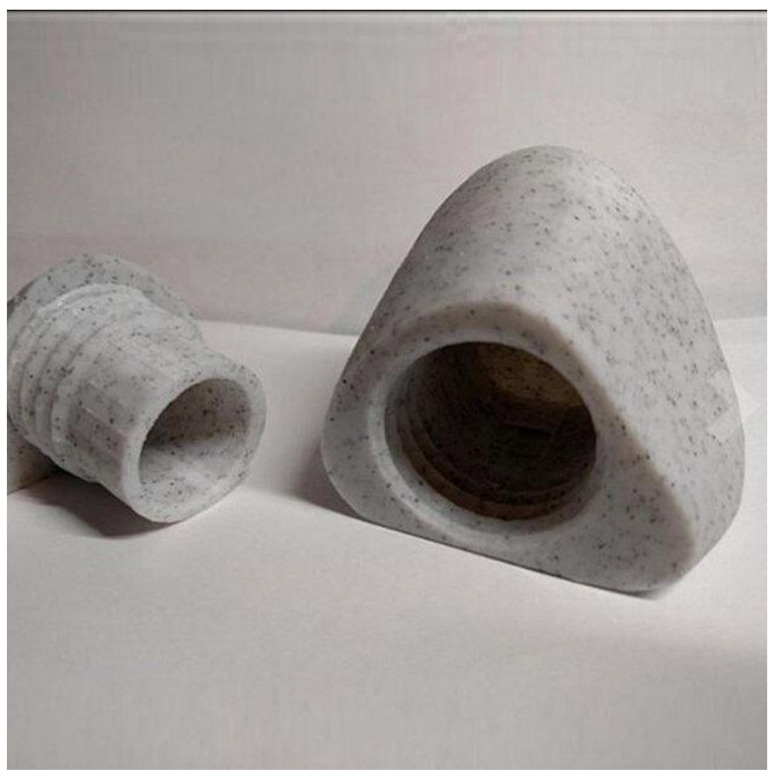
An image of 3D-printed items made out of ceramic-filled filament.

Ceramic-filled filaments can be utilized in various industries, such as aerospace, where they are used for high-temperature components such as engine parts and thermal shields. The automotive sector benefits from ceramic-filled filaments in the manufacturing of heat-resistant parts like brake components and engine mounts [116,117,118]. Figure 8 depicts an item being 3D printed out of ceramic-filled filament.

Thus, ceramic-filled filaments offer the ability to incorporate ceramics into 3D-printed objects, providing high-temperature resistance and enhanced mechanical properties. However, the brittleness of ceramic-filled objects should be considered, and careful attention must be given to printability to ensure successful prints.

### 2.8. Magnetic Filaments

Magnetic filaments, alternatively referred to as magnetically responsive filaments, represent a class of 3D printing raw materials that feature the capacity to manifest magnetic characteristics. These filaments are commonly composed of a thermo-plastic polymer that has been infused with magnetic particles, including but not limited to iron, ferrite, or neodymium. The inclusion of magnetic additives confers distinctive properties to the printed articles, facilitating their interaction with magnetic fields and affording a variety of applications in various domains [119].

The inclusion of magnetic particles in the filament matrix results in the manifestation of magnetic properties in the printed objects. The capacity of these entities to attract or repel magnetic fields confers upon them magnetic responsiveness, which can be exploited for the purposes of manipulation and control across diverse applications. The manipulation of magnetic characteristics can be achieved through the modulation of magnetic particle concentration and composition, thereby facilitating a diverse spectrum of magnetic intensities and dynamics [120].

The ability to 3D-print objects with inherent magnetic properties opens up numerous possibilities in fields such as robotics, sensing, and education. In robotics, magnetic filaments can be utilized to create custom magnetic grippers, actuators, or joints that enable the precise control and manipulation of objects. In sensing applications, these filaments can be employed to fabricate magnetic sensors, proximity switches, or even magnetic encoders for precise position detection [121].

The educational value of magnetic filaments is also noteworthy, as they offer a hands-on approach to learn about magnetism and its applications. Students can design and print objects that demonstrate magnetic behavior, allowing for interactive and engaging learning experiences [122].

However, it is important to note that magnetic filaments may pose challenges in terms of printability. The presence of magnetic particles can affect the flow and extrusion properties of the filament, requiring adjustments to printing parameters such as temperature and extrusion speed. Additionally, the orientation and alignment of the magnetic particles during printing can influence the magnetic behavior of the printed object, necessitating careful consideration during the design and printing process [123,124].

**Figure 9:**
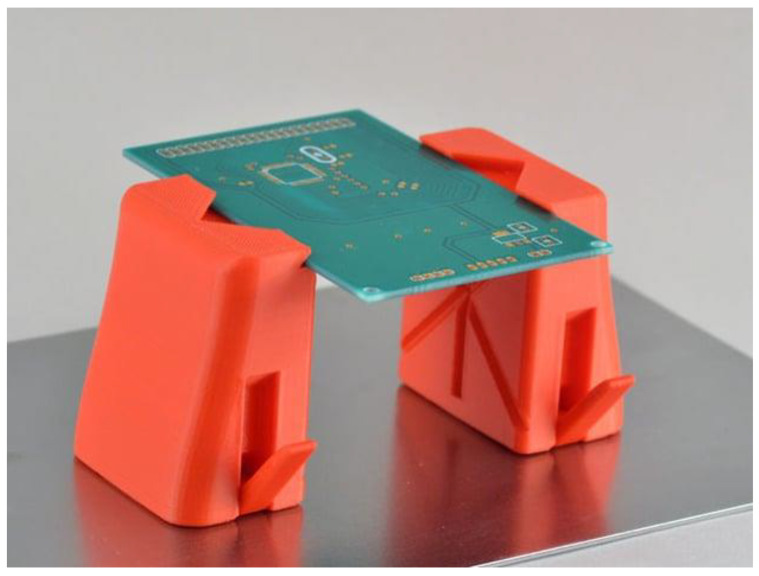
An image of 3D-printed items made out of magnetic filament [127].

The applications of magnetic filaments continue to expand as researchers and engineers explore new possibilities. In the medical field, these filaments show potential for creating magnetically responsive drug delivery systems, magnetic resonance imaging (MRI)-compatible devices, or even magnetic scaffolds for tissue engineering. In consumer electronics, magnetic filaments can be used to design and produce customized magnetic holders, cable management solutions, or sensor mounts [125,126]. Figure 9 depicts a 3D-printed item made out of magnetic filament.

In this context, magnetic filaments combine the versatility of 3D printing with the magnetic properties of the embedded particles, offering unique opportunities in robotics, sensing, education, and other fields. While challenges exist in terms of printability and particle alignment, ongoing research and development efforts are expanding the range of applications and optimizing the performance of magnetic filaments. As the field progresses, these filaments hold promise for the creation of innovative, magnetically responsive objects that enhance functionality and interaction in various domains.

### 2.9. Glow-in-the-Dark Filaments

The addition of glow-in-the-dark 3D printing filaments has been a noteworthy advancement in the field of additive manufacturing, as it has brought about advanced luminescent characteristics to the printed items. Such filaments are composed of phosphorescent materials that are designed to absorb and retain light energy, resulting in a bright luminescence in environments with low levels of light or complete darkness.

The manufacturing procedure of luminescent filaments entails the incorporation of phosphorescent pigments into fundamental materials, such as Polylactic Acid (PLA) or Acrylonitrile Butadiene Styrene (ABS), materials that are frequently utilized in 3D printing operations. The pigments consist mainly of strontium aluminate, which are supplemented with additional additives to augment their luminescent properties. The pigments have the ability to capture photons upon being exposed to light sources, and subsequently store the absorbed energy. This stored energy is then released as visible light when the luminosity is reduced [128].

The adaptability of luminescent filaments is a highly desired feature, as it enables the production of a diverse array of items, spanning from ornamental objects to practical constituents and prototyping substances. The precise calibration of printing parameters is imperative for the optimization of the performance of glow-in-the-dark filaments. The utilization of these filaments typically requires a moderate increase in temperature compared to standard filaments. Additionally, modifications to the printing speed and layer height are conducive to improved adhesion and print quality. In addition, the incorporation of printers featuring a heated platform serves as an efficient measure to minimize the possibility of warping that may occur during the printing procedure [129].

**Figure 10:**
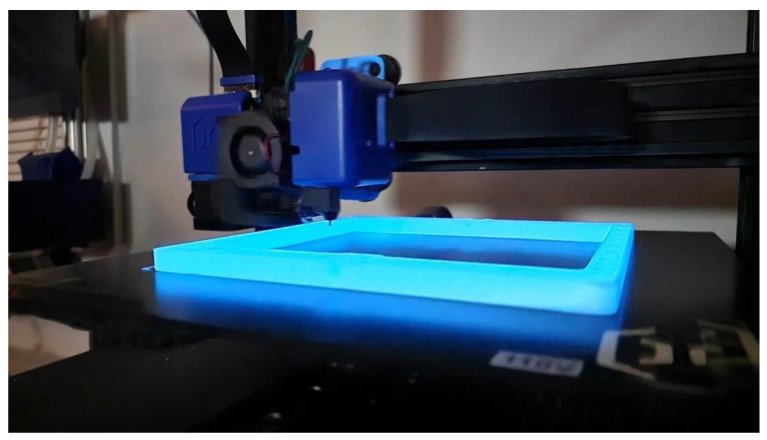
An image of 3D-printed items made out of glow-in-the-dark filament.

It is noteworthy that the luminescent characteristics demonstrated by these filaments may vary depending on the particular brand and composition utilized. Certain filaments exhibit a muted and understated luminescence, while others demonstrate a vivid and powerful radiance. The duration of the luminous effect is variable and dependent on the type of filament utilized, as some filaments exhibit a longer lifespan in comparison to others. Therefore, it is advisable to conduct a thorough examination of the product specifications and user feedback in order to choose a filament that corresponds to the intended luminosity and longevity [126,130,131]. Figure 10 depicts an item being 3D printed out of glow-in-the-dark filament.

To summarize, the introduction of glow-in-the-dark 3D printing filaments constitutes a noteworthy advancement in additive manufacturing, enhancing both the visual appeal and practicality of printed items. The incorporation of phosphorescent substances provides a captivating visual aspect to diverse applications, promoting ingenuity and aesthetic representation.

### 2.10. Stone-Filled Filaments

Stone-filled 3D printing filaments are composite raw materials consisting of PLA mixed with powdered stone, offering a unique matte and rough appearance that closely resembles authentic stone. These filaments possess a higher density compared to standard PLA filaments, contributing to the realistic feel and texture of the printed objects. The density also reduces the risk of warping during the printing process, ensuring more stable and accurate prints [132].

One notable feature of stone-filled filaments is the variation in color and texture that occurs naturally in the stone particles. Each print exhibits unique gradient color linings, further enhancing the realistic stone-like appearance. Moreover, different print settings, such as temperature and layer height, can be adjusted to achieve different finishes, allowing for customization and artistic experimentation [133].

However, it is important to consider the drawbacks of stone-filled filaments. One such drawback is their brittleness. The presence of powdered stone within the filament matrix reduces the overall flexibility and impact resistance of the printed objects. As a result, careful handling and post-processing are required to prevent breakage or damage.

**Figure 11:**
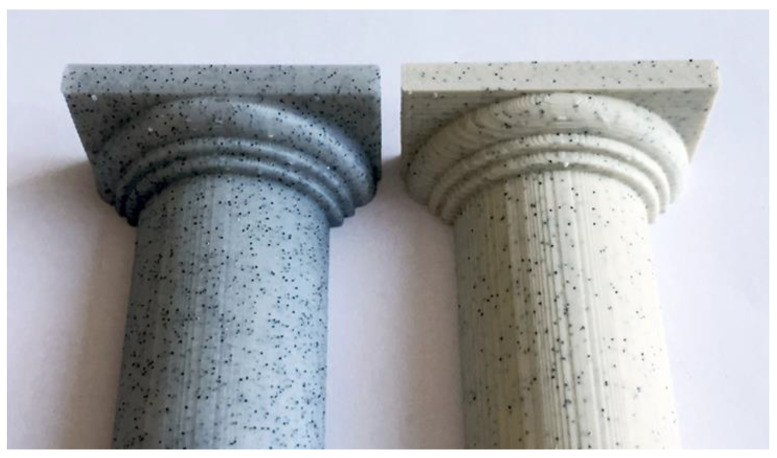
An image of 3D-printed items made out of stone-filled filament [135].

Another challenge associated with stone-filled filaments is their abrasive nature. The stone particles can cause increased wear on the printer nozzles due to their hardness and roughness. Regular maintenance and nozzle replacements may be necessary to mitigate the effects of abrasion and maintain print quality over time [134]. Figure 11 shows a 3D-printed item made out of stone-filled filament, while Table 1 depicts the mechanical properties of such filaments [134].

In conclusion, stone-filled 3D printing filaments provide a visually appealing and realistic option for achieving a stone-like finish in printed objects. The higher density and low risk of warping contribute to the quality and stability of the prints. However, the inherent brittleness and potential nozzle abrasion are important factors to consider when working with these filaments. With proper handling and maintenance, stone-filled filaments offer a compelling choice for creating 3D prints with a distinctive stone aesthetic [134,139,140,141].

## 3. Selection Criteria

### 3.1. Selection Criteria Based on Mechanical Properties

In the context of 3D printing potential projects requiring materials with advanced mechanical properties, several options from the aforementioned list stand out as suitable choices. Carbon fiber-filled filament, metal-filled filament, and glass fiber-filled filament are particularly noteworthy due to their enhanced mechanical properties. Such composite filament materials combine their elevated mechanical properties with the ability to fabricate intricate and complex geometries via 3D printing [142].

Carbon fiber-filled filament is widely recognized for its exceptional mechanical performance. The addition of carbon fiber reinforcement significantly enhances the filament’s strength, stiffness, and lightweight properties. The aligned carbon fibers contribute to increased tensile strength, rigidity, and resistance to deformation and fatigue. This material is commonly employed in aerospace, automotive, and engineering applications where structural integrity and weight reduction are paramount. Metal-filled filaments, featuring metallic particles within a polymer matrix, also offer compelling mechanical properties. These filaments exhibit improved thermal and electrical conductivity, as well as enhanced strength. While their mechanical properties may not be equivalent to fully dense metals, metal-filled filaments are extensively used for prototyping functional metal parts. Post-processing techniques such as polishing, plating, or sintering can be employed to further optimize the material’s mechanical performance.

Glass fiber-filled filament is another noteworthy option for applications requiring good mechanical properties. The incorporation of glass fibers significantly enhances the filament’s strength, stiffness, and dimensional stability. Glass fiber-filled filaments exhibit excellent tensile and flexural strength, making them suitable for applications where resistance to impact and bending loads is critical. These materials find applications in industries such as automotive, aerospace, and consumer goods, where mechanical strength and durability are essential.

Conversely, materials such as wood-filled, stone-filled, conductive, magnetic, and glow-in-the-dark filaments may be less suitable for projects prioritizing strong mechanical properties. While these materials offer unique properties such as aesthetics, conductivity, or magnetism, their mechanical strength may not be comparable to carbon fiber-, metal-, or glass fiber-filled filaments. As a result, they are more commonly utilized for artistic, decorative, or specialized functional purposes rather than for applications requiring high structural integrity or load-bearing capabilities.

To summarize, when selecting materials for a project that demands high mechanical properties in 3D printing, carbon fiber-filled, metal-filled, and glass fiber-filled filaments are recommended due to their excellent strength, stiffness, and performance characteristics. These materials offer superior mechanical properties compared to wood-filled, stone-filled, conductive, magnetic, and glow-in-the-dark filaments, which are better suited for applications where aesthetics, specialized functionality, or unique optical properties take precedence over mechanical strength.

### 3.2. Selection Criteria Based on Surface Finish

When considering a project that requires a material with a unique surface finish for 3D printing, each of the 10 aforementioned materials offers distinctive characteristics. However, certain materials stand out for their ability to provide exceptional surface finishes, while others may be less suitable in this regard.

Starting with carbon fiber-filled filament, it primarily excels in mechanical properties rather than surface finish. Although it offers a textured appearance due to the presence of carbon fibers, the surface may not possess the smoothness or lustrous finish desired for certain applications. Therefore, while carbon fiber-filled filament is exceptional for mechanical strength, it may not be the top choice when surface finish is the primary focus.

Metal-filled filament, on the other hand, offers intriguing possibilities for unique surface finishes. The presence of metallic particles within the filament can give rise to a metallic sheen or a textured appearance, depending on the specific metal used. Post-processing techniques like sanding, polishing, or chemical treatments can further enhance the surface finish, enabling a range of aesthetic possibilities. This makes metal-filled filaments well-suited for applications where a metallic appearance or texture is desired.

Wood-filled filament presents a distinctive surface finish, with visible wood fibers embedded within the printed object. This filament replicates the natural appearance and texture of wood, making it an appealing choice for applications requiring a rustic or organic finish. The printed objects exhibit the characteristic grain patterns and textures associated with wood, lending an authentic and unique surface finish.

Stone-filled filament offers a similar appeal, replicating the appearance and texture of stone materials. The printed objects exhibit a grainy and textured surface reminiscent of natural stone. This material can be particularly valuable for architectural models, sculptures, or designs that aim to capture the aesthetics and tactile qualities of stone.

Conductive filament, as the name suggests, possesses unique electrical conductivity properties rather than a distinctive surface finish. While it may not provide a specific surface appearance, it can be used to create functional objects requiring electrical conductivity, such as sensors, circuit components, or electromagnetic shielding. Magnetic filament, similarly, does not exhibit a particular surface finish but is designed to possess magnetic properties. Its primary function lies in applications where magnetic characteristics are required, rather than offering a distinct surface appearance.

Glow-in-the-dark filament also lacks a specific surface finish but is known for its ability to emit light in the dark. Objects printed with this filament can absorb and store light energy, emitting a soft glow when in a dark environment. This unique property is suitable for applications that require visibility in low-light conditions or for creating visually striking objects.

Finally, glass fiber-filled filament, although not renowned for its surface finish, offers improved mechanical properties and dimensional stability. It may not present a distinct visual appearance or surface texture, but it contributes to enhanced strength and rigidity, making it a valuable choice for applications where mechanical performance takes precedence over surface finish.

In summary, while materials such as metal-filled filament, wood-filled filament, and stone-filled filament can provide unique and aesthetically appealing surface finishes, carbon fiber-filled filament and glass fiber-filled filament may be less suitable when surface finish is the primary consideration. Conductive filament, magnetic filament, and glow-in-the-dark filament, although offering unique properties, may not exhibit specific surface finishes but are better suited for functional applications. It is important to consider the specific requirements of the project and the desired surface finish characteristics when selecting the appropriate material for 3D printing.

## 4. Discussion

Filled filaments as raw materials in FDM/FFF 3D printing technology exhibit a number of advantages when compared to “simpler” filaments consisting of solely one material. Filled 3D printing filaments are a type of 3D printing filament that are infused with different materials such as wood, metal, or carbon fiber. One of the main advantages of filled 3D printing filaments is that they can add unique properties to the final printed object, such as increased strength, durability, and stiffness. Additionally, these filaments can be more cost-effective than printing directly with metal, as they can mimic the properties of these materials while being less expensive. Another advantage is that these filaments can add aesthetic value to the final print, as the added materials can create a unique texture or appearance that cannot be achieved with traditional 3D printing filaments. Overall, filled 3D printing filaments offer a range of benefits that can enhance the functionality and appearance of the final printed object [143,144].

However, this kind of filament features a number of potential disadvantages that end-users must take into account before selecting it. Firstly, reduced print speed is one potential disadvantage of using filled 3D printing filaments. The added density of filled filaments can require the printer to work harder to extrude the material, which can result in slower print speeds and longer print times. This can be a particular concern when printing larger or more complex objects, as the increased print time can significantly impact the overall project timeline. To minimize this issue, it may be necessary to adjust printing settings or use a printer with a higher torque extruder to better handle the added density of filled filaments [145].

Secondly, another potential disadvantage of filled 3D printing filaments is the risk of clogging the printer nozzle. Some filled filaments contain particles, such as wood or metal fibers, that can become trapped in the nozzle and cause blockages, leading to failed prints and potential damage to the printer. To prevent clogging, it may be necessary to use a larger nozzle size or a specialized hot end designed to handle filled filaments. It is also important to regularly clean the nozzle and perform maintenance on the printer to ensure that any accumulated debris or particles are cleared away. In some cases, it may be necessary to use a different type of filament altogether to avoid the risk of clogging [142].

Also, limited availability is another potential disadvantage of filled 3D printing filaments. While traditional filaments such as PLA and ABS are widely available from many different suppliers, filled filaments may be more difficult to find, particularly if you need a specific type of material. This can be a particular issue for less-common materials, such as metal or wood-filled filaments, which may only be available from specialized suppliers. In some cases, it may be necessary to order the filament online or from a specific manufacturer, which can result in longer lead times and higher shipping costs. To avoid issues with availability, it may be necessary to plan ahead and order your filament well in advance of when you need it, or consider using a more readily available alternative filament if possible [146].

In addition, cost is another potential disadvantage of filled 3D printing filaments. Filled filaments can be more expensive than traditional filaments due to the added materials used to create the unique properties and appearance of the filament. Depending on the specific type of filled filament, the cost may be significantly higher than traditional filaments, which can make them less accessible to users with limited budgets. Additionally, the cost of filled filaments can vary widely depending on the supplier and material, making it important to shop around to find the best prices. While filled filaments may offer unique benefits that traditional filaments cannot match, the higher cost may be a limiting factor for some users [147,148].

Furthermore, another potential disadvantage of filled 3D printing filaments is the increased wear on printer components. Filled filaments can be more abrasive than traditional filaments due to the added particles or fibers, which can cause increased wear on the printer nozzle and other components. Over time, this increased wear can result in the need for more frequent repairs or component replacement, which can add to the overall cost of using filled filaments. To minimize wear on the printer components, it may be necessary to use a hardened or wear-resistant nozzle and to regularly inspect and clean the printer components. Additionally, it may be helpful to use filled filaments sparingly or to limit their use to specific applications to avoid excessive wear and tear on the printer [149,150].

An alternative to using a single filled filament material combining the matrix and the infused materials is multi-material Fused Deposition Modeling (FDM) processes. These processes empower the creation of intricate, multi-component objects that incorporate diverse material properties in a single print. The versatility of multi-material printing allows for the fusion of materials with distinct characteristics, such as flexible and rigid, conductive and non-conductive, or translucent and opaque, all within the same object. This capability is especially valuable for producing complex geometries and fully assembled parts without the need for extensive post-processing assembly, a boon for industries requiring lightweight, integrated designs, such as aerospace and automotive. However, it demands the careful consideration of material compatibility, extruder systems, support structures, and post-processing, alongside acknowledging the inherent challenges in this advanced printing technique.

The future of Fused Deposition Modeling (FDM) and Fused Filament Fabrication (FFF) printing holds promising directions that are poised to further revolutionize the industry. Firstly, a broader range of printable materials with enhanced properties, including improved strength, flexibility, and conductivity is anticipated. This includes an expansion of filled filament materials, such as carbon fiber, metals, and wood, offering even more diverse material choices and applications in industries like healthcare, aerospace, and electronics. Moreover, advancements in multi-material printing and support structures will continue to facilitate the creation of complex, fully assembled objects with ease. As automation and robotics intersect with 3D printing, we may see more autonomous 3D printers capable of managing multiple materials and tasks simultaneously. Additionally, sustainable and eco-friendly materials and recycling solutions will likely play a significant role in reducing the environmental impact of 3D printing processes. Finally, increased accessibility to FDM/FFF technology through cost-effective, desktop printers will empower individuals and small businesses to explore new creative and industrial possibilities, democratizing the manufacturing landscape.

## 5. Conclusions

This study explored the use of advanced composite materials in FDM/FFF 3D printing manufacturing processes, with a particular focus on filled filaments. In order to improve the mechanical, thermal, and functional properties of the printed parts, it was necessary to investigate the potential benefits and challenges associated with incorporating additives into the filaments. Through a comprehensive evaluation of the literature, it became clear that filled filaments offer promising opportunities for improving the performance of 3D-printed objects. The addition of fillers such as carbon fibers, glass fibers, nanoparticles, or other reinforcing agents can considerably improve the mechanical strength, stiffness, and impact resistance of 3D-printed components. Moreover, fillers can also enhance the thermal conductivity, flame retardancy, and electrical properties of the materials, thereby expanding their application scope.

However, it is important to acknowledge that the incorporation of fillers into filaments presents certain challenges. The selection of appropriate filler materials, their dispersion within the polymer matrix, and the optimization of printing parameters require careful consideration. The choice of filler content, size, shape, and distribution significantly influences the final properties of the printed parts. Additionally, compatibility issues between the fillers and the base polymer, as well as potential processing difficulties, must be addressed to ensure successful printing and optimal performance.

Despite these challenges, filled filaments have demonstrated their potential to revolutionize various industries, including aerospace, automotive, electronics, and biomedical sectors. The ability to tailor the properties of 3D-printed parts through the judicious selection and incorporation of fillers offers new avenues for customization, lightweighting, and functional integration. The advancements in material science and additive manufacturing technologies are continuously expanding the possibilities and driving the adoption of filled filaments in industrial applications.

Further research and development efforts are needed to overcome the current limitations and fully exploit the potential of advanced composite materials in FDM/FFF 3D printing processes. Future studies should focus on enhancing the understanding of filler–polymer interactions, optimizing processing parameters, and exploring novel filler materials. Additionally, the development of reliable models and simulation tools to predict the mechanical and functional properties of printed parts would be valuable for design and optimization purposes.

In conclusion, filled filaments hold great promise for advancing the capabilities of FDM/FFF 3D printing technology. By leveraging the unique properties of composite materials, it is possible to achieve enhanced performance, increased functionality, and expanded application possibilities. With continued research and development, filled filaments are poised to play a significant role in shaping the future of additive manufacturing, paving the way for innovative and sustainable solutions in various industries.

## Figures and Tables

**Table 1:** Mechanical properties of filled materials [134].

Material	Matrix Material	Heat Deflection Temperature (ISO 75) [136] (Avg. °C)	Impact Resistance (ISO 179-1) [137] (kJ/m^2^)	Tensile Strength (ISO 527-1) [138] (Mpa)
Wood-filled filaments	PLA	50 °C	19 kJ/m^2^	46 Mpa
Metal-filled filaments	PLA	53 °C	13 kJ/m^2^	23 Mpa
Carbon fiber-filled filaments	NYLON PA11	192 °C	30 kJ/m^2^	51 Mpa
Glass-filled filaments	NYLON PA11	186 °C	34 kJ/m^2^	62.8 Mpa
Conductive filaments	PLA	50 °C	18 kJ/m^2^	50 Mpa
Flexible/TPE (Thermoplastic Elastomer) filaments	TPU	52 °C	no break	13 Mpa
Ceramic-filled filaments	PLA	52 °C	15 kJ/m^2^	45 Mpa
Magnetic filaments	PLA	53 °C	12 kJ/m^2^	22 Mpa
Glow-in-the-dark filaments	PLA	50 °C	6 kJ/m^2^	55 Mpa
Stone-filled filaments	PLA	57 °C	2.9 kJ/m^2^	38 Mpa

## Data Availability

Not applicable.
